# Clinical outcomes and differential effects of PI3K pathway mutation in obese versus non-obese patients with cervical cancer

**DOI:** 10.18632/oncotarget.23664

**Published:** 2017-12-23

**Authors:** Perry Grigsby, Adnan Elhammali, Fiona Ruiz, Stephanie Markovina, Michael D. McLellan, Christopher A. Miller, Anupama Chundury, Ngoc-Anh L. Ta, Ramachandran Rashmi, John D. Pfeifer, Robert S. Fulton, Todd DeWees, Julie K. Schwarz

**Affiliations:** ^1^ Department of Radiation Oncology, Washington University School of Medicine, St. Louis, MO, USA; ^2^ Alvin J. Siteman Cancer Center, Washington University School of Medicine, St. Louis, MO, USA; ^3^ Division of Nuclear Medicine, Mallinckrodt Institute, Washington University School of Medicine, St. Louis, MO, USA; ^4^ Department of Radiation Oncology, MD Anderson Cancer Center, University of Texas Health Science Center, Houston, TX, USA; ^5^ McDonnell Genome Institute, Washington University School of Medicine, St. Louis, MO, USA; ^6^ Saint Louis University School of Medicine, St. Louis, MO, USA; ^7^ Department of Pathology and Immunology, Washington University School of Medicine, St. Louis, MO, USA; ^8^ Department of Obstetrics and Gynecology, Washington University School of Medicine, St. Louis, MO, USA; ^9^ Department of Cell Biology and Physiology, Washington University School of Medicine, St. Louis, MO, USA

**Keywords:** obesity, cervical cancer, PI3K, PTEN, mutation

## Abstract

The purpose of this study was to evaluate the effect of obesity and obesity-associated factors on the outcomes of patients with cervical cancer. Outcomes were evaluated in 591 patients with FIGO Ib to IV cervical cancer treated uniformly with definitive radiation. Patients were stratified into 3 groups based upon pretreatment Body Mass Index (BMI): A ≤ 18.5; B 18.6 – 34.9; and C ≥ 35. The 5-year freedom from failure rates were 58, 59, and 73% for BMI groups A, B, and C (p = 0.01). Overall survival rates were 50, 59, and 68%, respectively (p = 0.02). High expression of phosphorylated AKT (pAKT) was associated with poor outcomes only in non-obese patients. Obese patients with PI3K pathway mutant tumors had a trend toward favorable outcomes, while a similar effect was not observed in non-obese patients. Compared to similar tumors from non-obese hosts, *PIK3CA* and *PTEN* mutant tumors from obese patients failed to express high levels of phosphorylated AKT and its downstream targets. These results show that patients with obesity at the time of diagnosis of cervical cancer exhibit improved outcomes after radiation. PI3K/AKT pathway mutations are common in obese patients, but are not associated with activation of AKT signaling.

## INTRODUCTION

Increasing body mass index (BMI) has been associated with increased cancer-related mortality. Calle and colleagues found that overweight women had an increased risk of death from all causes and that the death rate in women from cervical cancer increased with increasing BMI [1]. In the UK’s Million Women cohort study, Reeves and associates found that cervical cancer incidence and mortality increased with increasing BMI [2]. However, Xu and colleagues reported results from the Cancer Genome Atlas (TCGA) showing lower mortality in cervical cancer patients with higher BMI values [3]. Although the analysis by Xu using TCGA data demonstrated lower mortality in cervical cancer patients with higher BMI values, these patients were treated with a variety of techniques and outcome data was not prospectively collected. The aim of the current study was to use a well-annotated clinical database to evaluate the association of obesity and cervix cancer patient outcomes after uniform treatment with curative intent radiation. A prospectively collected institutional tumor bank was used to determine whether obesity associated factors (metformin, insulin, and PI3K/AKT signaling) were associated with outcomes in the context of definitive radiation treatment.

## RESULTS

**Figure 1:**
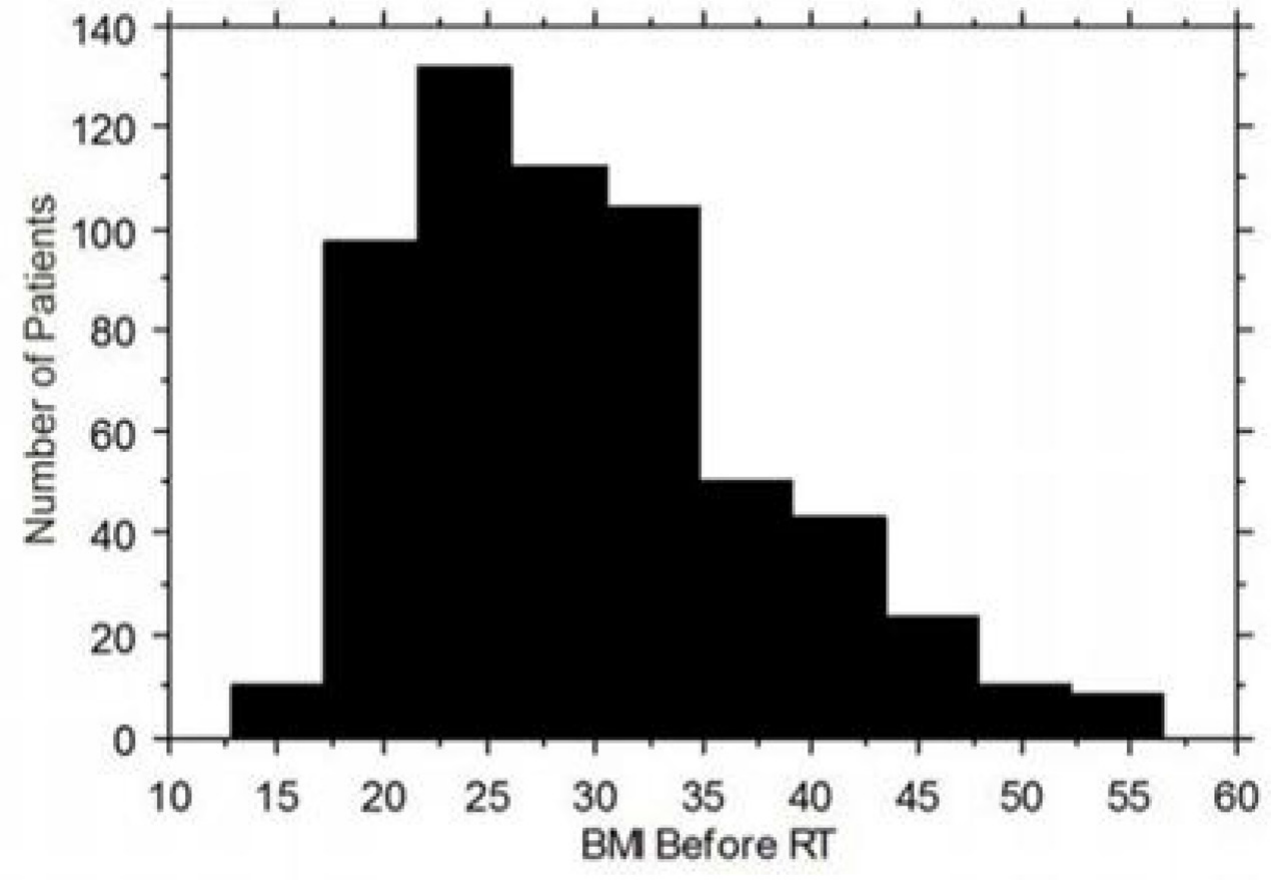
Patient characteristics and BMI distribution of the study population Pretreatment Body Mass Index (BMI) distribution histogram for 591 patients treated with radiation for cervical cancer.

Patient baseline characteristics are shown in Table 1. There were no significant differences in the traditional patient- and tumor-related prognostic factors of age, tumor stage, pretreatment ^18^F-fluoro-deoxy-glucose (FDG) positron emission tomography (PET) lymph node (LN) status and histology relative to the patient’s BMI. Patients with a high BMI were more likely to have Type II diabetes, take metformin, and take insulin. The distribution of patient BMI is shown in Figure 1. We have previously reported that cervical tumor FDG uptake quantified by Standardized Uptake Value (SUV) on pretreatment FDG-positron emission tomography (PET) scans is associated with poor prognosis [4]. To determine whether obesity remained a significant predictive factor in the setting of other known pretreatment risk factors, including cervical tumor SUV, a multivariate analysis was performed (Table 2). Cox proportional hazards modeling demonstrated that obesity remained significant in a model that included clinical stage, cervix tumor SUV and pretreatment lymph node status. There was no association between patient obesity and individual pretreatment risk factors including clinical stage, lymph node status and cervix tumor SUV. Similarly, there was no association between tumor human papilloma virus (HPV) status and patient obesity (data not shown).

**Table 1:** Patient baseline characteristics

	All N = 591	BMI A ≤ 18.5 N = 33	BMI B 18.6 – 34.9 N = 427	BMI C ≥ 35 N = 131	p-value
Age					
Range	23-92	32-81	23-92	24-83	N.S.
Mean	52	52	52	52	
Stage					
I (Ia1, Ia2, Ib1, Ib2)	200	9	131	60	N.S.
II (IIa, IIb)	237	13	175	49	
III (IIIa, IIIb)	139	9	110	20	
IV (IVa, IVb)	15	2	11	2	
BMI					
Range	13-57	13-18.5	18.6-34.9	35-57	N.A.
Median	27.5	18	26.1	40.5	
PET lymph nodes (LN)					
None	261	11	182	68	N.S.
Pelvis	240	18	178	44	
Pelvis & Para-artic	84	3	62	19	
P+P+SCV	6	1	5	0	
Histology					
Squamous	502	29	368	105	N.S.
Adenocarcinoma	60	2	37	21	
Other	29	2	22	5	
Metformin					
Yes	35	2	14	19	<0.0001
No	544	31	405	108	
Unknown	12	0	8	4	
Insulin					
Yes	30	1	12	17	<0.0001
No	549	32	407	110	
Unknown	12	0	8	4	
Type II					
Yes	75	2	33	40	<0.0001
No	504	31	386	87	
Unknown	12	0	8	4	

**Table 2:** Final results of proportional hazards model for recurrence

	Multivariate hazard ratio (95% CI)	P-value
Lymph Node Status (LN+ vs LN-)	1.692 (1.338, 2.320)	0.0010
Cervix Standardized Uptake Value (SUV)	1.020 (1.002, 1.037)	0.0280
Stage I (ref)		0.0018
Stage II	1.436 (0.982, 2.100)	
Stage III	2.117 (1.413, 3.171)	
Stage IV	2.630 (1.039, 6.658)	
BMI >= 35 vs BMI < 35	0.655 (0.439, 0.979)	0.0388

**Figure 2:**
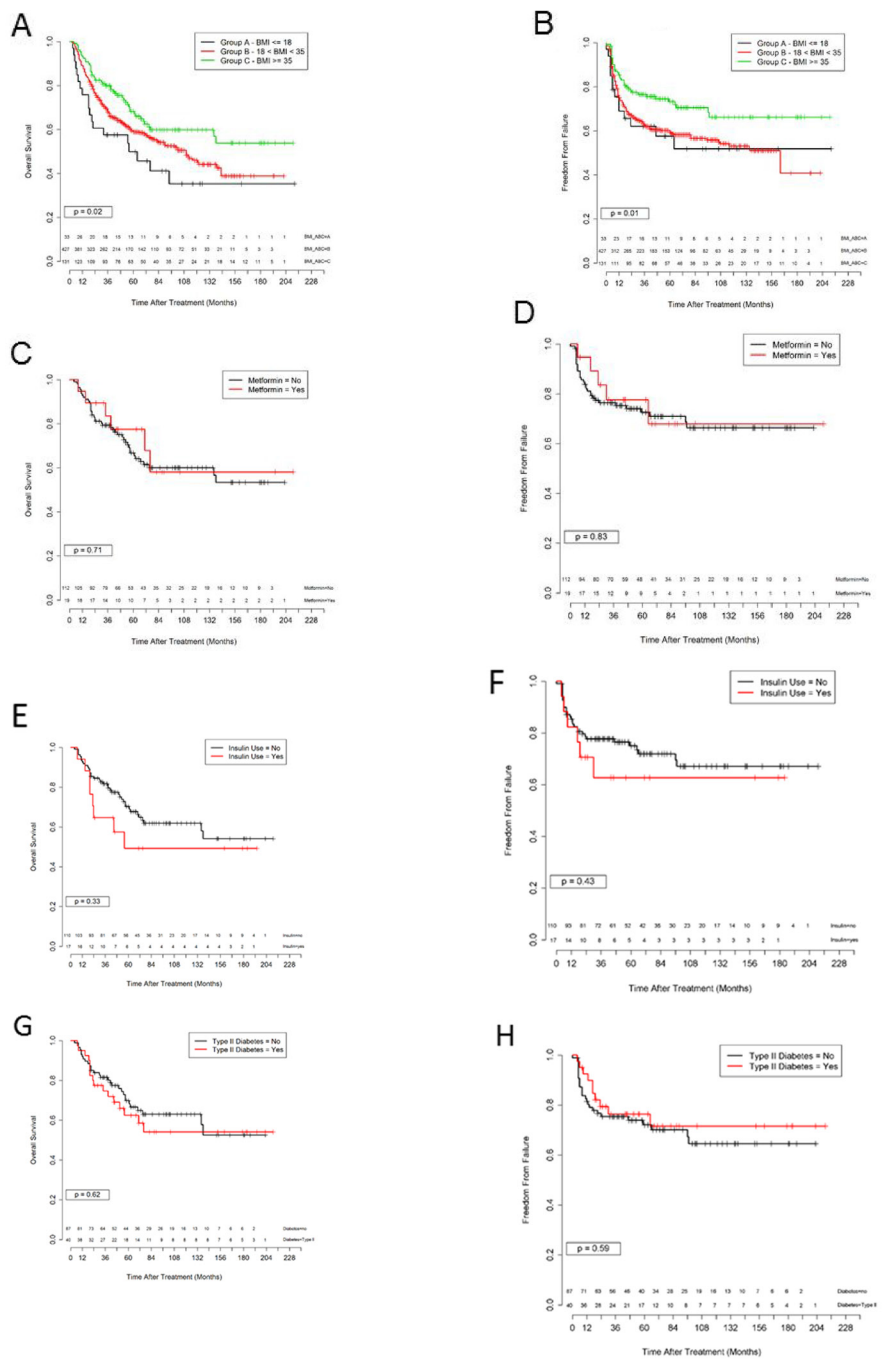
Survival outcomes separated by obesity and metformin use **(A)** Kaplan Meier curve for overall survival for patients in BMI group: A ≤ 18.5 (black); B 18.6 – 34.9 (red); and C ≥ 35 (green), p = 0.02. C. **(B)** Kaplan Meier curve for freedom from failure for patients in BMI group: A ≤ 18.5 (black); B 18.6 – 34.9 (red); and C ≥ 35 (green), p = 0.01. **(C)** Kaplan Meier curve for overall survival for patients from BMI group C with (red) and without (black) metformin use (p=0.71) **(D)** Kaplan Meier curve for freedom from failure for patients from BMI group C with (red) and without (black) metformin use (p=0.83). **(E)** Kaplan Meier curve for overall survival for patients from BMI group C with (red) and without (black) insulin use (p=0.33). **(F)** Kaplan Meier curve for freedom from failure for patients from BMI group C with (red) and without (black) insulin use (p=0.43). **(G)** Kaplan Meier curve for overall survival for patients from BMI group C with (red) and without (black) a diagnosis of diabetes (p=0.62). **(H)** Kaplan Meier curve for freedom from failure for patients from BMI group C with (red) and without (black) a diagnosis of diabetes (p=0.59).

Overall survival outcomes are shown in Figure 2A. Overall survivals at 5 years were significantly improved for obese patients treated with radiation (50, 59, and 68% for BMI groups A, B, and C p = 0.02). Corresponding 5-year FF rates were 58, 59, and 73% respectively (Figure 2B, p = 0.01). Metformin use was more common in BMI Group C (15%) than in Groups A (6%) or B (3%); however FF and OS were not affected by metformin use (Figure 2C and 2D). Similarly, FF and OS were not affected by diabetes or insulin use (Figures 2E-2H).

**Figure 3:**
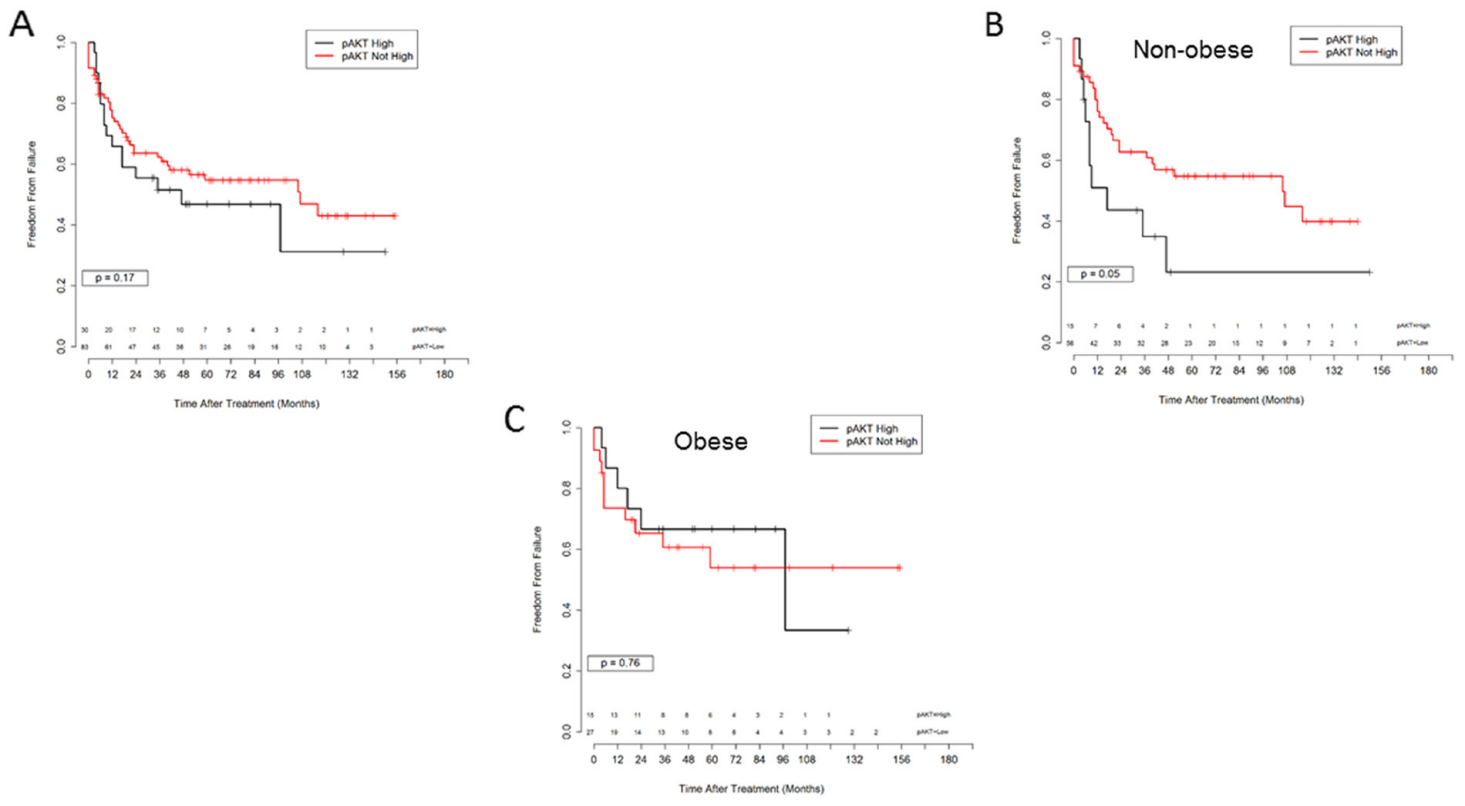
Immunohistochemistry for pAKT and outcome in obese versus non obese patients **(A)** Freedom from failure for all patients, high versus not high pAKT IHC, p = 0.17. **(B)** Freedom from failure, BMI ≤ 30, high versus not high pAKT IHC, p=0.05. **(C)** Freedom from Failure, BMI > 30, high versus not high pAKT IHC, p=0.76.

We have previously reported that increased expression of phosphorylated AKT is associated with poor prognosis after definitive radiation for cervical cancer [5]. To determine whether activation of AKT signaling was associated with outcome in obese versus non-obese patients, we analyzed expression of phosphorylated AKT (pAKT) by immunohistochemistry (IHC) using a commercially available antibody specific for AKT phosphorylated at serine 473 (S473). High pAKT expression by IHC was not associated with a statistically significant decrease in FF amongst all-comers (p=0.17, Figure 3A). High pAKT expression was associated with significantly reduced FF only in patients with BMI ≤ 30 (p=0.05, Figure 3B). In contrast, in the cohort of patients with BMI > 30, high pAKT expression was not associated with FF (p=0.76, Figure 3C). These results imply that the significance of pAKT expression as a biomarker for poor outcome after radiation treatment depends on patient obesity. For patients with a low BMI, expression of pAKT is predictive of poor outcome after irradiation. For obese patients, pAKT expression alone is not predictive, suggesting that downstream signals from AKT have less impact on radiation responses when the patient is obese and the resulting tumor environment is influenced by the obese state.

**Figure 4:**
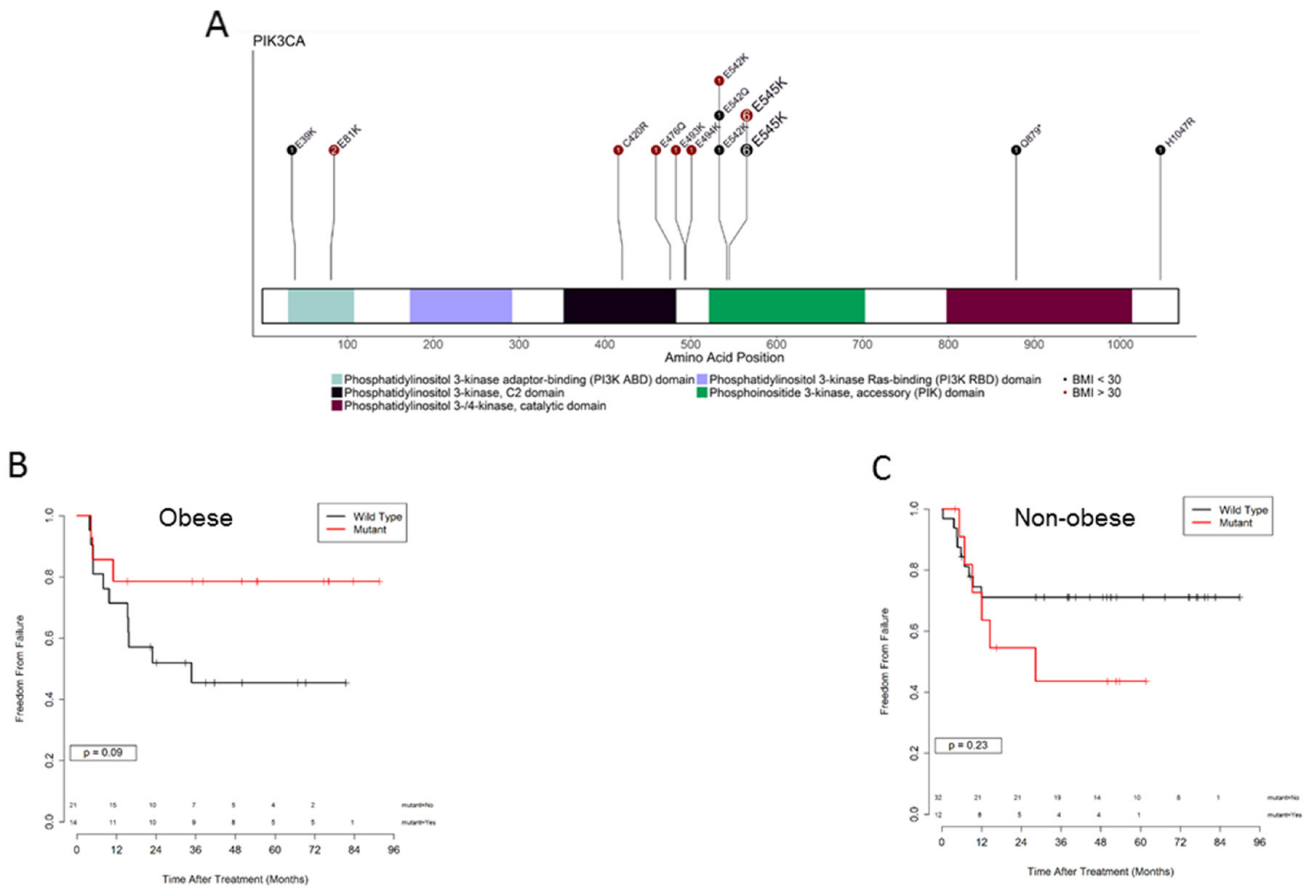
PI3K pathway mutations and outcome in obese versus non obese patients **(A)** Distribution of PIK3CA somatic mutations in obese and non-obese patients. **(B)** Kaplan Meier curves for freedom from failure in obese patients (BMI > 30) with (red) and without (black) *PIK3CA* and *PTEN* mutations, p =.09. **(C)** Kaplan Meier curves for freedom from failure in non-obese patients (BMI < 30) with (red) and without (black) *PIK3CA* and *PTEN* mutations, p = 0.23.

Somatic mutations in the PI3K/AKT signaling pathway have been identified in many cancers and are thought to promote inappropriate activation of AKT signaling. To identify somatic mutations in *PIK3CA* and *PTEN* (including *PTEN* copy number (CN) loss) in our patient population, targeted exome sequencing was performed on 91 pretreatment cervical tumor biopsies. The most common *PIK3CA* mutation was E545K in both groups (Table 3 and Figure 4A). *PIK3CA* and *PTEN* mutations were identified in 30 samples, 14 from non-obese patients and 16 from obese patients. Previous work from our group identified alterations in expression of genes from the PI3K/AKT pathway that were associated with incomplete metabolic response after chemoradiation in cervical cancer, and preliminary data demonstrated an association between *PIK3CA* activating mutations and inferior recurrence-free survival outcome after radiation [5, 6]. For this reason we compared FF outcomes for obese and non-obese patients with and without PI3K pathway mutations. There was a trend toward improved FF for obese patients with PI3K pathway mutations (p=.09) (Figure 4B). In contrast, non-obese patients with PI3K pathway mutations experienced similar if not inferior FF outcomes compared to non-obese patients without PI3K pathway mutations (p=.23) (Figure 4C).

**Table 3:** *PIK3CA*, *PTEN* and BMI in the WUSTL sequencing cohort

Sample	PIK3CA	PTEN	BMI	HPV subtype
705973	p.E545K	CN Loss	18.3	HPV 52
760832	p.E542K	WT	18.8	HPV 16
724788	p.E39K	WT	23.2	HPV 16, HPV 18
727879	p.E545K	WT	23.2	HPV 16
740838	p.E545K	WT	23.2	HPV 18
704786	p.E542Q	WT	23.7	HPV 16
707755	WT	CN Loss	24	HPV 16
734998	p.E545k, p.Q879^*^	WT	24.4	HPV 31
733024	p.H1047R	WT	25.2	HPV 16
703822	p.E545K	WT	25.5	HPV 16
722396	p.E545K	WT	25.5	HPV 16
710808	WT	CN Loss	26	Many
704849	WT	CN Loss	26.1	HPV 16
711380	WT	CN Loss	26.6	Negative
736133	p.E545K	WT	31.6	HPV 16
720170	WT	p.R233^*^	31.8	Negative
704821	p.E81K	WT	32.1	HPV 16
704956	p.E493K, p.E476Q, p.E494K	WT	35	HPV 58
728723	p.E81K	WT	35.7	HPV 16
720059	p.E545K	WT	37.3	Negative
714694	p.E545.K	WT	37.6	HPV 16
731976	WT	p.R130Q	39.1	HPV 16
733384	WT	CN Loss	39.7	HPV 33
714960	p.E542K	WT	39.9	HPV 16
736462	p.E545K	WT	43.2	HPV 16
711012	p.E545K	WT	43.5	HPV 16
738147	p.C420R	p.R130G, CN Loss	45	Negative
707913	WT	CN Loss	47.1	HPV 16
755439	p.E545K	WT	47.1	Many
746486	WT	p.T319fs	56.5	Negative

**Figure 5:**
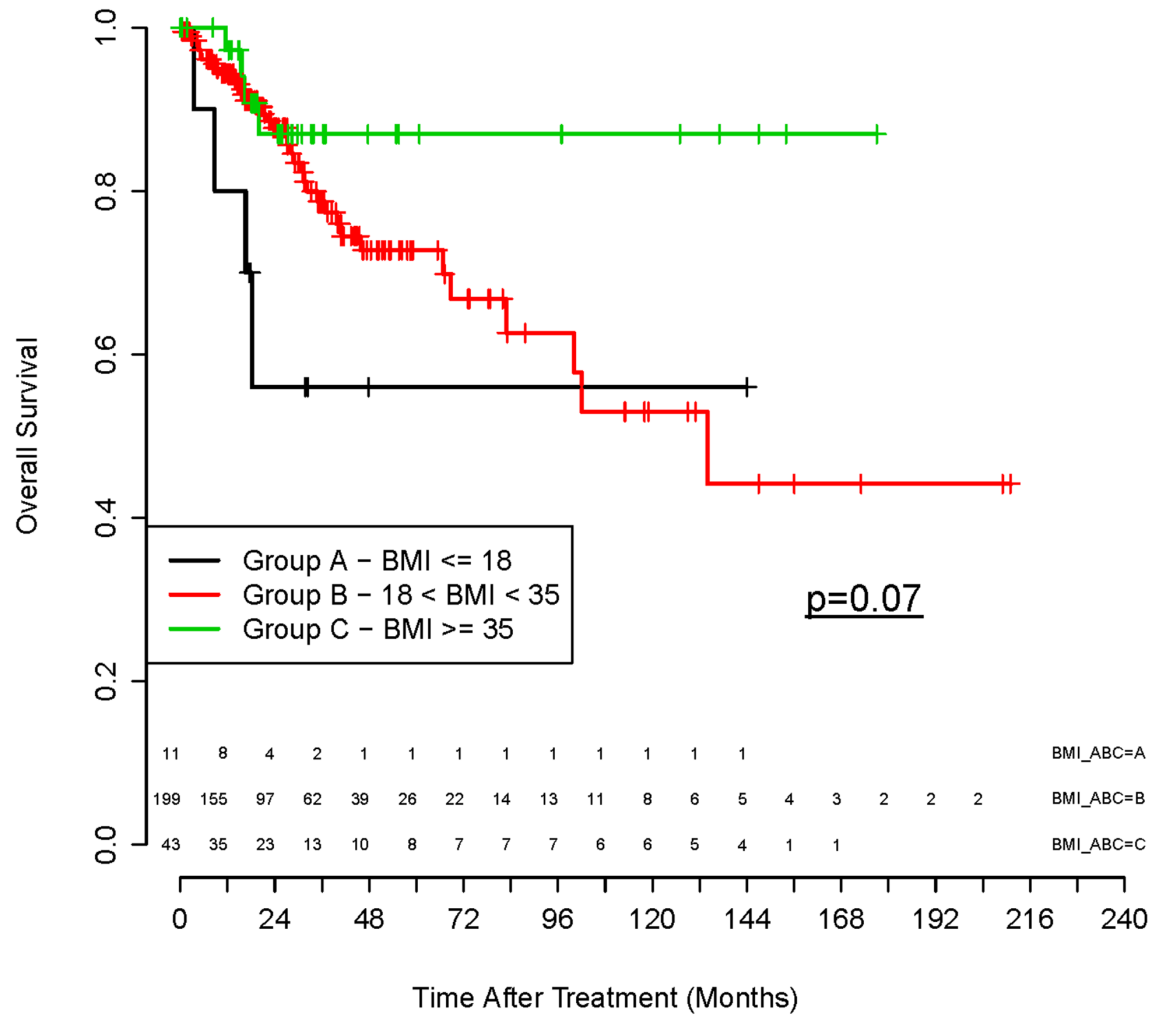
Overall survival outcomes from The Cancer Genome Atlas (TCGA) dataset separated by pretreatment BMI Overall survival outcomes obtained from the TCGA dataset based on pretreatment BMI: BMI group: A ≤ 18 (black); B 18 – 35 (red); and C ≥ 35 (green), p= 0.07.

**Figure 6:**
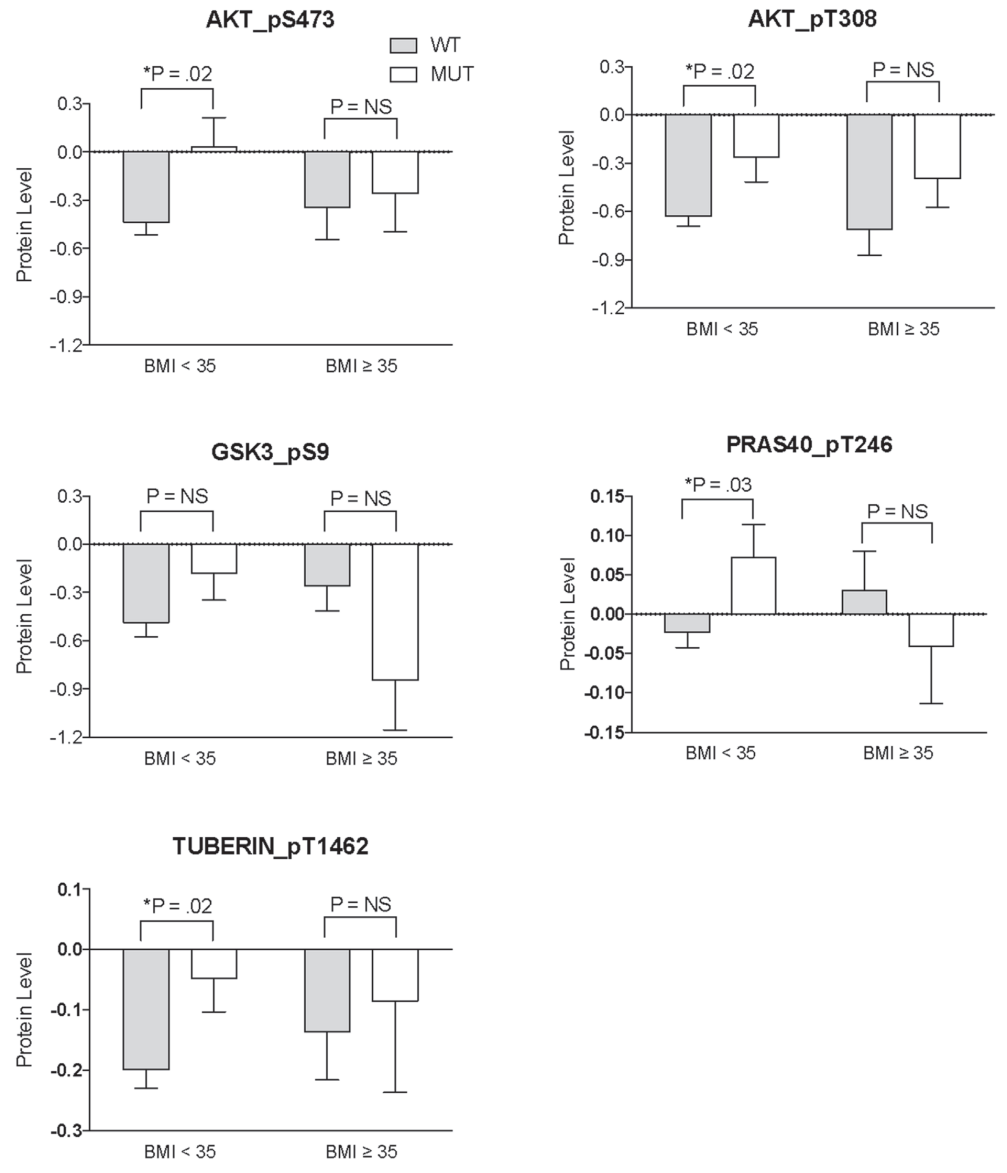
Phosphoprotein expression from PIK3CA and PTEN mutant tumors in obese versus non-obese hosts Log2 normalized phosphoprotein levels were compared for AKT_pS473, AKT_PT308, PRAS40_pT246, GSK_PS9 and Tuberin_pT1462 using a two tailed *t*-test with multiple comparison correction.

We then used reverse phase protein phosphorylation data from the Cancer Genome Atlas Project to test whether phosphorylation of AKT and AKT downstream targets was different in cervical tumors from patients who are obese versus non-obese. Among TCGA cervical cancer patients, obesity showed a trend toward improved overall survival, however this result did not reach statistical significance (p =.07, Figure 5). We then specifically examined whether protein phosphorylation patterns for AKT and downstream targets of AKT were different in *PIK3CA* and *PTEN* mutant tumors from obese versus non-obese hosts. In non-obese hosts, *PIK3CA* and *PTEN* mutant tumors displayed statistically significant increased phosphorylation of AKT at 2 key sites critical for full activation of AKT kinase activity, S473 and threonine 308 (T308) (Figure 6). In contrast, no increase in AKT phosphorylation at either site was observed for *PIK3CA* and *PTEN* mutant tumors from patients with BMI > 35. In addition, phosphorylation of PRAS40 and TUBERIN, two downstream direct targets of AKT kinase, was increased in *PIK3CA* and *PTEN* mutant tumors from non-obese hosts (p=.03 and p=.02, respectively), but phosphorylation of PRAS40 and TUBERIN was not increased in *PIK3CA* and *PTEN* mutant tumors from patients with BMI > 35. It should be noted, however, that not all AKT targets demonstrated differences in phosphorylation based upon obesity of the host (Figure 6 GSK3 and data not shown).

## DISCUSSION

In this study, we report that severely obese patients with cervical cancer treated with radiation have a better survival outcome than obese, normal, and underweight patients. Most investigators report that obese patients are at a higher risk for developing certain types of cancer and that their survival outcomes are poor when compared to normal weight patients. The mechanisms for poor outcomes in obese patients include insulin resistance, hyperinsulinemia, inflammatory responses, and increased bio-availability of steroid hormones. Our clinical data for cervical cancer are unique in that obese patients, specifically severely obese patients (BMI > 35) treated with definitive radiation, have favorable survival outcomes after treatment. Our study represents the largest reported series to date of clinical outcomes related to obesity for cervical cancer patients.

Metformin use has been reported to both decrease cancer risk and decrease cancer mortality. In our study, the severely obese women with cervical cancer, outcomes were not affected by metformin use, type II diabetes, or insulin use. Thus, the favorable outcome results in severely obese patients cannot be explained by diabetes or metformin use. Therefore, we explored other potential biological explanations for the difference in survival outcomes related to obesity.

Previous work from our group identified alterations in expression of genes from the PI3K/AKT pathway that were associated with incomplete metabolic response after chemoradiation in cervical cancer, and preliminary data demonstrated an association between *PIK3CA* activating mutations and inferior disease-free survival outcome after radiation [5, 6]. Examination of the specific mutations present in obese and non-obese patients (Table 3) demonstrated roughly equal numbers of *PIK3CA* activating and *PTEN* inactivating mutations between the two groups. The most common *PIK3CA* mutation was E545K in both groups. E545K is a common cancer-associated mutation in the helical domain of the p110alpha subunit of PI3K, which results in constitutive PI3K activity and has been reported to transform cells and enhance tumorigenic phenotypes [7–9]. A number of less well characterized mutations in *PIK3CA* were identified in our data set, including C420R, which has been reported to induce oncogenic transformation by promoting membrane binding of p110alpha [10]. The most frequent mechanism of *PTEN* mutation was *PTEN* copy number deletion. *PTEN* R130Q/G point mutations and a single frameshift at T319 were identified in the obese patient group.

Although obese patients had high rates of *PIK3CA* and *PTEN* mutations, obesity was not associated with increased expression of pAKT, raising the possibility that *PIK3CA* and *PTEN* mutations in obese patients do not activate downstream signaling via AKT to the same extent as similar mutations in non-obese patients. Increased expression of pAKT was associated with inferior outcome after chemo-radiation only in the non-obese group, suggesting that AKT downstream signals are differentially regulated between the two groups. To further evaluate potential molecular mechanisms for the observed difference in outcome for PI3K mutant tumors in obese versus non-obese patients, we compared publically available reverse phase protein array (RPPA) data from The Cancer Genome Atlas Project (TCGA). In non-obese hosts, *PIK3CA* and *PTEN* mutant tumors displayed significantly higher levels of phosphorylation of AKT at S473 and T308, key sites critical for full activation of AKT kinase activity. In contrast, no increase in AKT phosphorylation at either site was observed for *PIK3CA* and *PTEN* mutant tumors from patients with BMI > 35. Phosphorylation of downstream AKT targets PRAS40 and TUBERIN was increased in *PIK3CA* and *PTEN* mutant tumors from non-obese hosts, but phosphorylation was not increased in *PIK3CA* and *PTEN* mutant tumors from patients with BMI > 35. These results suggest that *PIK3CA* and *PTEN* mutations more effectively activate AKT kinase activity and phosphorylation of select AKT downstream targets in cervical tumors when the patient is non-obese.

It is important to note that in the TCGA project for cervical cancer, the majority of patients were treated with surgery for early stage disease. When we compared survival outcome data from the TCGA based on pretreatment BMI groups, we noted a similar trend of improved survival outcome with obesity, however outcomes in the TCGA cohort did not reach statistical significance (p =.07, Figure 5). There are several potential explanations for the lack of statistical significance in the TCGA cohort including smaller cohort size, shorter median follow-up time and non-uniform treatment. The effects of obesity on outcome may be different for patients who are managed by primary surgery versus radiation. For example, post-surgical complications may be increased in obese patients and this may obscure any protective effects on survival outcome. Alternatively, the protective effects of obesity on clinical outcomes may be more pronounced when patients are treated with primary radiation therapy. Additional study with prospectively collected clinical outcome databases will be needed to address this question.

While activation of the PI3K/AKT pathway is common in many cancers, and preclinical evidence for an oncogenic role for *PIK3CA* mutations is well accepted, mutations in *PIK3CA* have performed poorly as biomarkers for outcome in solid tumors [11]. Our study suggests that obesity may be one factor that influences AKT signaling downstream of an activating *PIK3CA* mutation. *PIK3CA* and *PTEN* genomic mutations may be more accurate biomarkers for cervical cancer outcome only for patients who are non-obese. These results could be used to inform future clinical trial design. Stratification on the basis of PI3K pathway mutation may be more effective if the host environment is considered.

The implications of obesity and its effects on signaling at the molecular level are complex. How an obese state influences cervical tumorigenesis and the response to anti-cancer treatments, including irradiation, are incompletely understood. Our data suggest that cervical tumor cell signaling through the PI3K/AKT pathway is distinct in obese versus non-obese patients and that these differences are associated with the response to concurrent standard of care chemoradiation. Additional preclinical study is needed to understand how the obese state may influence oncogenic signaling and radiation responses.

## MATERIALS AND METHODS

### Patient databases

**1) Clinical outcomes database (N=591)** Patients in the study cohort consisted of 591 patients with a new diagnosis of advanced cervical cancer seen at our institution from June 1997 to June 2014. Pre-treatment BMI (calculated using the National Institute of Health online calculator) was recorded for all patients. All patients underwent a pre-treatment workup including history and physical, examination under anesthesia, and a whole-body FDG-PET/CT.

**2) Immunohistochemistry cohort (N=113)** Archived formalin-fixed, paraffin-embedded (FFPE) specimens were used to construct a tissue microarray. Prospective data collection, retrospective data collection, and use of FFPE specimens were performed with University IRB approval with waiver of informed consent (201603148, 201108070, 201201099, 201208101, 201104085, and 201601055).

**3) Sequencing cohort (N=91)** A subset of 91 patients were prospectively enrolled into a tumor banking study at the time of initial diagnosis. This study was approved by the Institutional Review Board and all patients provided informed consent for sequencing (201105374). Tumor biopsies and blood were obtained prior to treatment and stored at the Tissue Procurement Facility.

### Radiation treatment

Definitive radiation with curative intent was administered in all (N=591) patients. Radiation treatment consisted of both external beam radiotherapy and intracavitary brachytherapy using techniques previously described [12]. The median prescribed external irradiation dose to the pelvic lymph nodes was 50.4 Gy. Concurrent chemotherapy (once weekly 40 mg/m^2^ cisplatin) was administered in 88% (N=518) patients.

### Statistical analysis

Overall survival (OS) and freedom from failure (FF) were the primary endpoints of the study. Survival outcomes were measured from the completion of treatment. Failure was defined as cancer recurrence anywhere within the body, both locally within in the pelvis and distantly (sites outside of the radiotherapy field). BMI groups were determined by using existing clinical categories of obesity as defined by the World Health Organization. Outcome-oriented methods for determining cutpoints proposed by Contal & O’Quigley were then used to determine groups within our patient population. [13] For analysis of all patients (n=591), patients were stratified into 3 BMI groups: A ≤ 18.5; B 18.6 – 34.9; and C ≥ 35. For the subset of patients undergoing mutational and IHC analysis, patients were stratified into only 2 groups (BMI less than or equal vs greater than 30) because of the smaller total number of patients in this subset. There were insufficient numbers of translational correlates in our institutional datasets from patients with BMI > 35 to provide meaningful statistical analysis. SAS v9.4 and R v3.0.3 were used for the analyses. P < 0.05 was set as the threshold for significance for all study outcomes. The Kaplan-Meier (product-limit) method and the Log-Rank test were used to derive time-to-event estimates and test for significance. Fisher’s Exact test was utilized to compare differences between categorical data. ANOVA and Independent *t*-tests were utilized to compare continuous covariates and z-test for proportions was used to test differences in proportions. Multivariate proportional hazards modeling was performed as previously described [14]. The final model that was constructed consisted of variables clinical stage, lymph node status and cervical tumor SUV from the pretreatment FDG-PET and BMI > 35.

### Immunohistochemistry analysis

A tissue microarray (TMA) was constructed from pre-treatment formalin-fixed paraffin embedded (FFPE) tumor specimens. Punches were taken from marked areas for tumor content and used to construct a tissue microarray on MTA-1 Manual Tissue Arrayer (Beecher Instruments, Inc., Sun Prairie, WI). Slides were prepared from 0.6μm sections, deparaffinized and rehydrated per manufacturer’s protocol. After antigen retrieval, slides were stained with anti-phospho-AKT antibody (Rabbit anti-human AKT-1 (phospho-S473) polyclonal, Spring Bioscience) per manufacturer’s protocol using the Ventana BenchMark ULTRA autostainer (Ventana Medical Systems Inc., Tuscon, AZ) using cell conditioning step CC1. Slides were scanned and digitized on ScanScope XT (Leica Biosystems, Buffalo Grove, IL), and analyzed using the web-based Aperio eSlide Manager platform (Leica Biosystems, Buffalo Grove, IL). Staining was reviewed in a blinded fashion by two reviewers, and given a consensus score for intensity of pAKT staining as absent, weak, intermediate or strong intensity.

### *PIK3CA* and *PTEN* sequencing

Tumor biopsies were sectioned and subjected to review by a pathologist. Only tumor specimens with ≥ 60% neoplastic cellularity and <20% necrosis were used for further analysis. DNA was extracted from frozen tumor tissue using QIAamp DNA kit (Qiagen, USA) according to the manufacturer’s instructions. Illumina libraries were constructed with dual-index barcode sequences and enrichment was performed for target regions as previously described [15]. Hybridization capture sequence data from tumor and matched normal DNA was used to catalogue somatic mutations in *PIK3CA* and *PTEN*, including loss of heterozygosity (LOH). More specifically, sequence data were aligned to GRCh37-lite_WUGSC_variant_2 (http://genome.wustl.edu/pub/reference/GRCh37-lite_WUGSC_variant_2/) using bwa-mem (Processing Profile c3e6b636310547caaa9776e9aca5e4c5: bwamem-stream 0.7.10 [-t 8]). Bams were merged using Picard 1.113 then deduplicated using Picard 1.113 api v6. Putative somatic point mutations were detected using tumor and normal Samtools r982 [16], Varscan 2.3.6 [17], Strelka 1.0.11, [18] and Somatic Sniper [19]. Putative somatic indels were identified using GATK (gatk-somatic-indel 5336) [20], Pindel 0.5 [20] and Varscan 2.3.6. Variants were merged and filtered as previously described. In addition, putative variants were filtered to remove known germline dbSNPs (dbSNP 137), artifacts detected in a panel of normal samples [21] variants with less than 8 reference-allele-supporting reads in the Normal DNA, and less than two supporting reads or a less than of 10% variant allele fraction in the tumor. *PTEN* LOH predictions were made using VarScan 2.3.6 based on read coverage distribution differences between tumor and matched normal using methods previously published [17]. Mutation locations were mapped to a protein framework using the lolliplot function in GenVisR package (http://bioconductor.org/package/GenVisR) implemented in R3.3.0.

### Reverse phase protein array data analysis

Reverse Phase Protein Array (RPPA) Data was generated by the Cancer Genome Atlas (TCGA) Research Network [22]. Normalized level 4 RPPA data from cervical cancer were obtained from the Cancer Proteome Atlas [23]. Clinical data including weight, height, and survival was obtained from cbioPortal [24, 25]. Log2 normalized protein levels were compared using a two tailed *t*-test with multiple comparison correction (Benjamini and Hochber alogrithm) using Matlab (MATLAB and Bioinformatics Toolkit 2015b, The MathWorks, Inc., Natick, Massachusetts, United States).

## Abbreviations

BMI: Body Mass Index
FDG: ^18^F – fluoro-deoxy-glucose
FF: freedom form failure
FIGO: International Federation of Gynecology and Obstetrics
HPV: human papilloma virus
IHC: immunohistochemistry
LN: pretreatment lymph node status
MUT: mutant
OS: overall survival
pAKT: phosphorylated AKT
PET: positron emission tomography
RPPA: reverse phase protein array
RT: radiation
SUV: standardized uptake value
TCGA: the Cancer Genome Atlas
TMA: tissue microarray
WT: wild type
